# Malignant and Nonmalignant Gene Signatures in Squamous Head and Neck Cancer

**DOI:** 10.1155/2012/752860

**Published:** 2012-04-12

**Authors:** Maria J. Worsham, Mei Lu, Kang Mei Chen, Josena K. Stephen, Shaleta Havard, Vanessa P. Schweitzer

**Affiliations:** ^1^Department of Otolaryngology-Head & Neck Surgery, Henry Ford Health System, Detroit, MI 48202, USA; ^2^Department of Biostatistics and Research Epidemiology, Henry Ford Health System, Detroit, MI 48202, USA

## Abstract

Genetic events specific to the pathogenesis of malignancy can offer clues to the tumorigenesis process. The objective of this study was to identify gene alterations that differentiate tumor and nontumor lesions in squamous head and neck cancer (HNSCC). DNA from 220 primary HNSCC with concurrently present tumor and nontumor lesions from the same patient was interrogated for genomic alterations of loss or gain of copy. Conditional logistic regression dealt with tumor and non-tumor records within a patient. Of 113 genes, 53 had univariate effects (*P* < 0.01), of which 16 genes remained in the multivariable model with *P* < 0.01. The model had a C-index (ROC) of 0.93. Loss of *CDKN2B* and gain of *BCL6, FGF3*, and *PTP4A3* predicted tumor. Loss of *BAK1* and *CCND1* and gain of *STCH* predicted nontumor. This highly powered model assigned alterations in 16 genes specific for malignant versus nonmalignant lesions, supporting their contribution to the pathogenesis of HNSCC as well as their potential utility as relevant targets for further evaluation as markers of early detection and progression.

## 1. Introduction

Knowledge of the genetic mechanisms that drive cancer growth and development is important in understanding the pathogenesis of malignancy and provides insights into the tumorigenesis process. The underlying hypothesis is that behavior of tumor cells is determined by genetic changes that alter cell growth, cell differentiation, programmed cell death, and cell migration. Cancer is the result of transformation from a normal to a malignant cell that results from accumulated mutations. Acquisition of a fully malignant phenotype in colon cancer is thought to occur because of multiple steps whose targets are alterations of growth-promoting oncogenes and growth-inhibiting cancer suppressor genes. The evolution in transformation from a normal squamous epithelial cell to a cancer cell is likewise assumed to require several steps, some defined by genetic alteration.

 Genetic alterations provide means of identifying tumor cells as well as defining changes that presumably determine biological differences from their normal counterparts. Chromosome aberrations have served as landmarks to identify cancer genes in many tumor types; however, individual gene loci altered in tumors cannot be deduced solely from the type of chromosome rearrangement [1]. Historically, the molecular pathogenesis of cancer has been teased out one gene at a time. Recent high-throughput genomewide candidate strategies such as the multiplex ligation-dependent probe amplification (MLPA) assay [2] to identify specific genes for gain and loss concurred with chromosomal aberrations and provide a novel index to estimate the extent of genomic abnormality with disease progression [1].

 Molecular genetic prognosticators can influence prevention, diagnosis, appropriateness of adjuvant chemotherapy, and, possibly, the chemotherapeutic regimen of cancer patients. Dissecting out processes specific to the pathogenesis of malignancy can distill key genetic biomarkers of HNSCC etiology, transformation, and progression.

 In this study, in a primary HNSCC cohort of 220 patients, with both tumor and nontumor lesions within a biopsy (tumor and nontumor from the same patient), we examined gene alterations of loss and gain to derive multivariate predictive models to discriminate malignant from nonmalignant lesions.

## 2. Methods

### 2.1. Patient Cohort

 Cohort subjects were male and female patients 18 years and older who underwent a HNSCC tissue biopsy at the Henry Ford Health System from 1986–2006. The use of formalin-fixed paraffin-embedded tissue blocks from patients with both tumor and nontumor records within the same biopsy and the collection of related patient information were approved by the Henry Ford Health System Institutional Review Board (IRB) Committee.

 In the event a study subject had more than one eligible biopsy over the course of the study period, the primary biopsy was the index biopsy and the pathology report date marked the study enrollment (index) date. Cohort subjects missing biopsy tissue blocks or insufficient tissue for molecular analysis were excluded.

### 2.2. Histopathology

 Pathology review of paraffin-embedded tissue sections captured all lesion types in a biopsy to include normal squamous epithelium, squamous dysplasia whether classified as mild, moderate, or severe/carcinoma in situ, and tumor. Severe dysplasia and carcinoma in situ lesions were grouped with tumor and considered as malignant outcomes. 

### 2.3. Molecular Analysis

#### 2.3.1. Processing Lesion Specimens for Molecular Analysis

 DNA was obtained from either whole 5 micron tissue sections (if the tissue block contained predominantly tumor or nontumor tissue) or from microdissected tissue as previously described [3]. Briefly, concurrently present tumor (severe dysplasia, carcinoma in situ, tumor) and nontumor (normal, mild/moderate dysplasia) lesions in the same paraffin-embedded formalin fixed tissue block were marked by the pathologist and individual lesions were microdissected from 5 micron sections mounted on glass slides using a single-use disposable scalpel blade under a dissecting microscope. This procedure minimizes mixing of normal and tumor subpopulations and yields lesion and tumor samples estimated to be at least 90% free from contamination with normal cells [3, 4].

#### 2.3.2. The Multiplex Ligation-Dependent Probe Amplification (MLPA) Assay

 MLPA has several advantages. It establishes the copy number of up to 41 nucleic acid sequences in one single reaction. MLPA probes are able to discriminate between sequences that differ in only one nucleotide. Moreover, MLPA reactions require a minimum of only 20 ng human DNA making it especially amenable for PCR of DNA from formalin-fixed paraffin-embedded head and neck tissues [3–5].

 Starting with, approximately, 20–50 ng of genomic DNA, for each subject in the cohort, DNA from tumor and nontumor is amplified for 122 probes (113 unique genes associated with cancer including HNSCC) as separate sets of three reactions (probe sets p005, p006, p007, MRC Holland, Amsterdam). Briefly, DNA, diluted with water to a total volume of 5 *μ*L, is denatured and fragmented by heating for 5 minutes at 98°C in a thermocycler. Binary MLPA probes are added and allowed to hybridize to their targets during an 16 hr incubation at 60°C, followed by the addition of dilution buffer and a ligase enzyme (MRC Holland, Amsterdam). During the 15-minute incubation at 60°C, the two parts of a probe become ligated to each other and become an amplifiable molecule if the complementary sequence is present in the DNA sample. This is followed by the addition of PCR primers, dNTPs and Taq polymerase, followed by the following cycles: one at 1 min 95°C, 10 cycles: 30 sec 95°C, 30 sec 70°C, 1 min 72°C; 30 cycles at 30 sec 95°C, 30 sec 60°C, 1 min 72°C. The same primer pair, one of which is tagged with a fluorescent dye, amplifies all (ligated) probes. The relative amounts of PCR product obtained reflect the relative amounts of ligated probes at the start of the PCR reaction. Amplification products are analyzed on a DNA sequencer (Applied Biosystems, Foster City, Ca), quantified and interpreted as previously described [1, 3, 4, 6–8].

### 2.4. Statistical Analysis

 Conditional logistic regression modeling was used to address tumor and nontumor lesions within the primary biopsy in an HNSCC patient. Analysis began by testing individual genes as risk predictors/discriminators for tumor and nontumor (univariate analysis). Genes with individual risks in a univariate analysis (*P* < 0.01) were candidates for the first multivariable model. Prior to multivariable modeling, genes were evaluated for their correlation and missing values. Highly correlated genes (correlation coefficient [*r*] > 0.7) or genes with larger missing values (>5%) were fitted separately along with other uncorrelated (*r* < 0.7) genes. The stepwise model selection was considered. The final model included genes with *P* < 0.01 along with odds ratios for loss or gain as risk predictors. The C-index/ROC (the receiver operating characteristic {ROC} curve), in a range of 0 to 1, is a measure of the model's predictive ability, where 0.5 indicates no discrimination and 0.7 or greater indicates that the model is predictive.

## 3. Results and Discussion

### 3.1. Results

 Matched tumor and nontumor lesions within each patient in the 220 primary HNSCC cohort comprised a total of 1076 tissue records. There were 504 normal/mild/moderate dysplasia lesions (495: normal squamous epithelium, 6: mild dysplasia, 3: moderate dysplasia), and 572 tumor lesions (568: tumor, 1: severe dysplasia, 3: carcinoma in situ (CIS)). Squamous mucosal dysplasia whether classified as mild, moderate, or severe/carcinoma in situ is considered intraepithelial neoplasia, and, as a precursor lesion group is separate from normal and tumor. The number of precursor, lesions is very small (13) in comparison to normal epithelium (495) and carcinomas (568). Of the 572 tumor lesions, 193 (34%) were laryngeal, 170 (30%) were oral cavity, 151 (26%) were pharyngeal (oropharyngeal/hypopharyngeal), and 58 (10%) were lesions in the other category (nasopharynx, nasal cavity, paranasal sinuses).

 The 1076 lesions were distributed among 932 tissue blocks, of which 434 (47%) had tumor lesions only, 363 (39%) had nontumor lesions only, and 135 (14%) tissue blocks had both tumor and nontumor lesions. Within a patient, the number of nontumor lesions ranged from 1 to 6 and from 1 to 7 for tumor lesions. Of the 220 patients with both tumor and nontumor within the same patient, 35 (16%) had 1 tumor and nontumor record, 28 (13%) had 2 tumor and nontumor records, 27 (12%) had 3 tumor and nontumor records.

 Fifty percent of cohort (110/220) subjects were Caucasian American (CA), 38% (84/220) were self-reported as African American (AA), 4% (9/220) were non-CA/non-AA (Hispanic: 2, Asian Pacific Islander: 2, Middle Eastern: 3, other: 2), and, in 8% (17/220), race was missing. Of the 220 patients, 25% (55/220) were female and 75% (165/220) were male.

 The missing value for each gene was in a range of 0% to 4.2%. Of the 113 unique genes (selected based on their association with cancer including HNSCC), 53 genes had univariate effects (*P* < 0.01) and were considered as the candidate genes for multivariable analyses. After the stepwise model selection, 16 genes remained in the multivariable model (*P* < 0.01) (Table 1). The model had a C-index (receiver operating characteristic (ROC)) of 0.93.

 The 16 genes in the final model with alterations of loss and/or gain accounted for loci along 7 chromosomes: 3, 4, 6, 8, 9, 11, and 21 (Table 1). Of these, 50% were altered in both tumor and nontumor, with loss or gain reflective of chromosomal aneuploidy. This copy number instability favored loss of *CDKN2A* (9p21), *CTNNB1 *(3p21), *IL2 *(4q26), *LTA* (6p21.3), and *TFF1* (21q22.3) in tumor, with corresponding gain in nontumor lesions, and gain of *FGFR1* (8p21), *MYC* (8q24.12), and *PRKDC* (8q11) in tumor, with corresponding loss in nontumor (Table 1).

 Loss of *CDKN2B* (9p21) and *LMO2* (11p13) and gain of *BCL6* (3q27), *FGF3* (11q13), and *PTP4A3* (8q24.3) predicted tumor. Loss of *BAK1* (6p21.3) and *CCND1* (11q13) and gain of *STCH* (21q11.1) predicted nontumor (Table 1).

 Analysis excluding the 6 mild dysplasia and 3 moderate dysplasia lesions from the nontumor group and the 1 severe dysplasia lesion and 3 CIS from the tumor group generated an identical multivariable model outcome.

### 3.2. Discussion

 Cancerous tissue in most cases has a distinctive appearance under the microscope. Distinguishing traits include a large number of dividing cells, variation in nuclear size and shape, variation in cell size and shape, loss of specialized cell features, loss of normal tissue organization, and a poorly defined tumor boundary. Biopsy and microscopical examination can also distinguish malignancy, precursor lesions of carcinoma in situ, mild, moderate, and severe dysplasia, and less reliably, hyperplasia, from normal appearing tissue.

 In the multistep process of tumorigenesis, hyperplasia, which refers to tissue growth based on an excessive rate of cell division, leading to a larger than usual number of cells but with a normal orderly arrangement of cells within the tissue, and considered reversible, is thought to precede dysplasia. Dysplasia, an abnormal type of excessive cell proliferation characterized by loss of normal tissue arrangement and cell structure, may revert to normal behavior, but, occasionally, these lesions gradually become malignant. Distinguishing true precursor lesions on the basis of morphology alone is often unreliable. In the molecular progression of HNSCC, normal or minimal dysplasia often harbors abnormal genotypes [9], which do not necessarily correlate with observable changes in phenotype (morphology) [10, 11].

 In this study, 16 gene alterations with significant discriminatory ability differentiated malignant HNSCC from nonmalignant tissue. For matched tumor and nontumor lesions from the same patient within the 220 primary HNSCC study cohort, examined for alterations in 113 unique genes with association to head and neck cancer, molecular alterations in “normal” appearing epithelium within the environment of a malignant biopsy harbored genotypic abnormalities that set them apart from malignant tissue.

 The 16 genes in the final model span loci along 7 chromosomes: 3p21:* CTNNB1*, 3q27:* BCL6*; 4q26: *IL2*; 6p21.3: *BAK1* and *LTA*; 8p12:* FGFR*, 8q24.12:* MYC*, 8q24.3:* PTP4A3*; 9p21: *CDKN2A*, *CDKN2B*; 11p13: *LMO2*, 11q13: *CCND1*, *FGF3*; 21q11.1: *STCH*, 21q22.3: *TFF1*. Gene alterations at these loci restate reported cytogenetic [7, 12–22] and molecularly altered regions by LOH and array CGH studies in HNSCC [1, 5, 7, 23–28]. Additionally, copy number loss in tumor and corresponding gain in nontumor, and vice versa, advocate aneuploidy events. The latter are highlighted for loss of *CDKN2A* (9p21), *CTNNB1 *(3p22), *IL2 *(4q26), *LTA* (6p21.3), and *TFF1* (21q22.3) in tumor, with corresponding gain in nontumor lesions, and gain of *FGFR1* (8p12), *MYC* (8q24.12), and *PRKDC* (8q11) in tumor, with corresponding loss in nontumor. Chromosomal instability occurs early along the tumorigenesis continuum and aneuploidy at the 9p21 locus affecting corresponding loss and gain in tumor and normal tissue, respectively, concurs with the proposed postulated model of molecular carcinogenesis for HNSCC [29].

 In HNSCC, chromosomal aberrations on the long arm of chromosome 3, resulting in gain of distal 3q segments, have been reported as recurring karyotypic alterations [22]. Gain of 3q is supported by increased copy number (3-4 copies) of *PIK3CA* at 3q26.3, *MME *(3q25.1), and *BCL6* genes at 3q27 [1] in HNSCC. In this study, gain of *BCL6* was significantly associated with tumor lesions. The protein encoded by *BCL6 *is a zinc finger transcription factor and acts as a sequence-specific repressor of transcription.

 Another chromosome 3 gene, *CTNNB1 *(catenin beta-1) in the short arm at 3p21, is an adherens junction protein, closely associated with adhesion, invasion, and metastasis in different types of tumors, including SCC of the tongue [30]. The 3p21 region had the highest rate of allelic deletion (63%) in HNSCC [31] and is supported by loss of *CTNNB1* in tumor lesions in this study. Corresponding gain of *CTNNB1* copy number in nontumor lesions underscores chromosomal instability and ensuing aneuploidy as early events in the tumorigenesis process.

 Loss and corresponding gain of *IL2* at 4q26 was significantly associated with tumor and nontumor lesions, respectively. The IL2 protein is produced by T cells in response to antigenic or mitogenic stimulation and is required for T-cell proliferation and other activities crucial to regulation of the immune response.

*BAK1* (6p21.3) is a proapoptotic member of the *BCL-2* family of genes that are involved in regulation of programmed cell death, and its increased expression had poorer disease-specific survival in oral tongue squamous cell carcinomas [32]. As a corollary to increased expression [32], in this study, loss of *BAK1* was a nontumor-specific event.

 Gene alterations were noted for 4 genes on chromosome 8, three on 8q and one on 8p. Gains or amplifications involving chromosome arm 8q are one of the most recurrent chromosomal alterations in head and neck tumors. The human protein tyrosine phosphatase type IVA, member 3, also known as *PTP4A3*, is located at 8q24.3 [33]. The protein encoded by this gene is a cell signaling molecule that participates in every aspect of cellular physiologic and pathologic processes [33]. Recent studies [34, 35] suggest that an excess *PTP4A3* may play a key role in the acquisition of metastatic potential of tumor cells. This study further supports gain of *PTP4A3* as a malignancy-associated alteration [36] in HNSCC.

The *MYC* oncogene, located at 8q24.12, encodes a transcription factor that plays a key role in cell proliferation, differentiation, and apoptosis [37]. Gain of *MYC* was significant for laryngeal tumor progression [38], and the concomitant over expression of *MYC* and *p53* oncogenes had worse disease-free survival suggesting a role for *p53* and *MYC* genes in progression of HNSCC [39]. In this study, gain of *MYC* significantly discriminated tumor from nontumor tissue. The corresponding loss of *MYC* copy number in nontumor suggests aneuploidy as a likely destabilizing event.

 DNA double-strand breaks repair pathway has been implicated in maintaining genomic integrity via suppression of chromosomal rearrangements.* PRKDC *(protein kinase, DNA-activated, catalytic polypeptide) is associated with chromosomal instability with risk of breast and uterine cervix cancer [40]. In this study, genomic instability at the 8q11 locus favored gain of *PRKDC *copy number in tumor and corresponding loss in nontumor.

*FGFR-1*, located at 8p21, had gain of copy number in tumor and corresponding loss in nontumor.* FGFR-1* expression has been detected in thyroid carcinoma [41] and in oral squamous cell carcinomas (OSCC). Amplification of *FGFR1* detected by FISH analysis on OSCC tissue microarray sections contributed to oral carcinogenesis at an early stage of development [42].

 Genetic alterations at the 9p21 locus have been linked to malignant progression in HNSCC [43, 44]. *CDKN2A *and* CDKN2B* genes map to 9p21 and are in tandem, spanning a region of approximately 80 kb [45, 46]. Inactivation of the *CDKN2A* (p14), *CDKN2B *(p15), and *CDKN2A* (p16) genes is a frequent event in human oral squamous cell carcinomas [47]. The main modes of *p*16^INK4a^ inactivation in HNSCC are known to include homozygous deletions (40–60%), mutations (15–20%), and gene hypermethylation events (5%) [47–49]. This study supports loss of *CDKN2A* and *CDKN2B* genes as independent predictors of tumor in HNSCC patients.

 Amplification of the 11q13 amplicon is driven by multiple genes, rather than only one or two genes at this site [50–52]. In this study, four genes at the 11q13 locus were interrogated, *CCND1*, *FGF3*, *EMS1*, and *RELA*, of which, gain of *CCND1*, *FGF3*, and *EMS1* were univariate (*P* < 0.0001) predictors of tumor. Multivariate analysis (*P* < 0.01) retained gain of *FGF3* in tumor and loss in nontumor, supporting involvement of amplification/gain of copy number of this gene in HNSCC [53, 54]. *FGF3* belongs to the basic fibroblast growth factor (FGF) gene family with a role in several important cellular processes, including regulation of cell growth and division, determination of cell type, formation of blood vessels, wound healing, and embryo development. In HNSCC, *FGF3* had a significantly higher frequency of amplification in hypopharyngeal tumors [55]. Loss of *CCND1 *was significantly associated with nontumor lesions in this study cohort, suggesting genomic instability/chromosomal aneuploidy events in the direction of corresponding gain of *CCND1* in tumor lesions (*P* < 0.001, univariate analysis). Overexpression and/or amplification of *CCND1* is reported in 35%–65% of patients with HNSCC and is associated with poor prognosis [56–58]. Its expression is deregulated in preinvasive lesions adjacent to invasive tumors and is associated with increased chromosomal instability and the likelihood of subsequent gene amplification. [59, 60] Loss of *CCND1* copy number in nontumor tissues in this study may reflect very early genomic instability at this chromosomal locus and supports reports of *CCND1* deregulation in preinvasive lesions of the upper aerodigestive tract with associated increased risk for the development of cancer accompanied by histologic progression during and after chemopreventive intervention [61, 62]. Though corresponding gain of *CCND1* was not retained in the final multivariate model, 24% of tumor lesions had copy number gain. Loss of *LMO2* (also known as *RBTN2* and *TTG2*), located at 11p13, predicted tumor lesions. This gene encodes a transcriptional cofactor critical for the development of hematopoietic stem cells [63].

 Gain of copy number at two 21q loci, *TFF1 *(21q22.3) and *STCH* (21q11.1), predicted nontumor lesions, and corresponding loss of *TFF1* was a significantly associated with tumor lesions. Loss of *TFF1* in tumor lesions with corresponding gain in nontumor supports genomic instability as a concerted early tumorigenesis event. *STCH* (stress 70 protein chaperone), at 21q11.1, a member of the heat shock protein 70 (HSP70) superfamily with cell-protective functions, was previously identified as a candidate gene for susceptibility to stomach cancer by genetic analyses [64]. *STCH *copy number gain in nontumor lesions remained in the final model as an independent predictor of nonmalignant tissue (corresponding loss in tumor lesions remained a univariate variable, *P* < 0.001).

 The model's discriminatory abilities (C-index/ROC of 0.93) support molecular distinctiveness of malignant versus nonmalignant tissue with significant predictive power. The latter is of particular significance because normal samples from patients with head and neck cancer, especially in the neighborhood of the tumor, can be genetically altered (field cancerization). The proximity of tumor and nontumor lesions, therefore, makes it harder to discriminate between these two entities. However, the relatively large number of tissue records (*n* = 1,076) from 220 patients was a factor in overcoming these challenges to yield a robust model with excellent ability (C-index = 0.93) to discriminate malignant and nonmalignant tissue within the same patient.

 Genetic alterations at 16 chromosomal loci underscore the association of already known genes as well as newer gene targets in HNSCC pathogenesis. The sixteen gene predictors spanning loci along 7 chromosomes cover an array of essential functions that ensure normal homeostasis to include DNA repair (*PRKDC*), initiation of carcinogenesis (*TFF1*), immune surveillance (*IL2*, *LTA*), cell cycle regulators (*CDKN2A*, *CDKN2B*), apoptosis (*BAK1*, *STCH*), regulation of cell proliferation and differentiation (*CCND1*, *FGF3*, *MYC*), transcription factors (*BCL6*), stem cell hematopoiesis (*LMO2*), adhesion, invasion and metastasis (*CTNNB1*, *FGFR1*), and acquisition of metastatic potential of tumor cells (*PTP4A3*), implicating these genes as key players in the tumorigenesis continuum.

## 4. Conclusion

 Genomic instability, a hallmark of malignant transformation, promotes a wide range of mutations, including chromosome deletions, gene amplifications, translocations, and polyploidy [40]. In this study, the directional loss and gain for several genes underscored the contribution of aneuploidy in early HNSCC tumorigenesis. Our data support distinct genetic signatures that discriminate malignant and nonmalignant tissue in HNSCC. The 16 gene alteration signature in this study suggests finely choreographed genomic instability events to achieve biological distinctiveness, providing clues to the drivers in invasive cancers as well as insight into gene rearrangements that might arise in nonmalignant lesions. The gene sets meet statistical rigor to segregate malignant squamous carcinoma lesions from nonmalignant lesions, providing an opportunity for researchers to investigate these cancer-associated genes as potential targets of therapy either as single targets or as sets of targets when these occur in the same cancer lesion.

## Figures and Tables

**Table 1:** Genes alterations that predict malignant (M, bolded) and nonmalignant (NM, italicized).

Effect	Chromosome	Odds ratio estimate	Lower CL*	Upper CL
^§M^ *CTNNB1* loss versus normal	3p22	**2.682**	**1.394**	**5.162**
^ NM^ *CTNNB1 *gain versus normal		*0.323*	*0.147*	*0.71*
*BCL6 *loss versus normal	3q27	0.55	0.27	1.12
^ M^ *BCL6 *gain versus normal		**8.989**	**3.155**	**25.612**
^§M^ *IL2 *loss versus normal	4q26	**3.697**	**1.774**	**7.705**
^ NM^ *IL2 *gain versus normal		*0.149*	*0.055*	*0.407*
^ NM^ *BAK1 *loss versus normal	6p21.3	*0.262*	*0.103*	*0.666*
*BAK1* gain versus normal		0.438	0.192	0.999
^§M^ *LTA *loss versus normal	6p21.3	**2.156**	**1.172**	**3.965**
^ NM^ *LTA *gain versus normal		*0.266*	*0.108*	*0.655*
^§NM^ *FGFR1 *loss versus normal	8p21	*0.275*	*0.126*	*0.598*
^ M^ *FGFR1* gain versus normal		**5.555**	**1.689**	**18.267**
^§NM^ *PRKDC* loss versus normal	8q11	*0.276*	*0.11*	*0.692*
^ M^ *PRKDC* gain versus normal		**5.449**	**2.09**	**14.206**
^§NM^ *MYC *loss versus normal	8q24.12	*0.221*	*0.097*	*0.503*
^ M^ *MYC *gain versus normal		**2.218**	**1.136**	**4.332**
*PTP4A3* loss versus normal	8q24.3	0.493	0.214	1.135
^ M^ *PTP4A3* gain versus normal		**12.158**	**3.461**	**42.71**
^§M^ *CDKN2A* loss versus normal	9p21	**1.845**	**1.013**	**3.362**
^ NM^ *CDKN2A* gain versus normal		*0.14*	*0.056*	*0.35*
^ M^ *CDKN2B* loss versus normal	9p21	**3.256**	**1.676**	**6.325**
*CDKN2B* gain versus normal		1.168	0.442	3.087
^ M^ *LMO2* loss versus normal	11p13	**4.977**	**2.16**	**11.466**
*LMO2* gain versus normal		0.573	0.205	1.607
*FGF3 *loss versus normal	11q13	0.882	0.447	1.741
^ M^ *FGF3* gain versus normal		**7.819**	**3.286**	**18.604**
^ NM^ *CCND1* loss versus normal	11q13	*0.403*	*0.22*	*0.736*
*CCND1* gain versus normal		1.239	0.634	2.421
*STCH* loss versus normal	21q11.1	1.788	0.833	3.839
^ NM^ *STCH *gain versus normal		*0.124*	*0.043*	*0.359*
^§M^ *TFF1 *loss versus normal	21q22.3	**3.019**	**1.514**	**6.02**
^ NM^ *TFF1 *gain versus normal		*0.08*	*0.024*	*0.268*

*CL: confidence limit, ^§^: genes with loss and gain signifying aneuploidy.
